# A cardiac-rehab behaviour intervention to reduce sedentary time in coronary artery disease patients: the SIT LESS randomized controlled trial

**DOI:** 10.1186/s12966-024-01642-2

**Published:** 2024-08-19

**Authors:** Sophie H. Kroesen, Bram M. A. van Bakel, Marijn de Bruin, Arzu Günal, Arko Scheepmaker, Wim R. M. Aengevaeren, Frank F. Willems, Roderick Wondergem, Martijn F. Pisters, Francisco B. Ortega, Maria T. E. Hopman, Dick H. J. Thijssen, Esmée A. Bakker, Thijs M. H. Eijsvogels

**Affiliations:** 1 Department of Medical BioSciences, Radboud University Medical Center, Geert Grooteplein Zuid 10, P.O. Box 9101, Nijmegen, 6500 HB The Netherlands; 2 Department of IQ healthcare, Radboud University Medical Center, Geert Grooteplein Zuid 10, Nijmegen, 6525 GA The Netherlands; 3 Department of Cardiology, Bernhoven Hospital, Nistelrodeseweg 10, Uden, 5406 PT The Netherlands; 4 Department of Cardiology, Rijnstate Hospital, Wagnerlaan 55, Arnhem, 6815 AD The Netherlands; 5 Department of Rehabilitation, Physical Therapy Science and Sport, University Medical Centre Utrecht Brain Centre, Utrecht University, Universiteitsweg 100, Utrecht, 3584 CG The Netherlands; 6 Research Group Empowering Healthy Behaviour, Department of Health Innovations and Technology, Fontys University of Applied Sciences, Rachelsmolen 1, Eindhoven, 5612 MA The Netherlands; 7 Centre for Physical Therapy Research and Innovation in Primary Care, Julius Health Care Centres, Universiteitsweg 100, Utrecht, 3584 CG the Netherlands; 8 Department of Physical Education and Sports, Sport and Health University Research Institute (iMUDS), University of Granada, Parque Tecnologico de la Salud, Av. del Conocimiento, s/n, Granada, 18007 Spain; 9 CIBERobn Physiopathology of Obesity and Nutrition, Institute of Health Carlos III, Av. Monforte de Lemos, 3-5. Pabellón 11. Planta 0, Madrid, 28029 Spain; 10 Faculty of Sport and Health Sciences, University of Jyväskylä, Keskussairaalantie 4, Jyväskylä, 40600 Finland; 11 Research Institute for Sports and Exercise Sciences, Liverpool John Moores University, Tom Reilly Building, Byrom Street, Liverpool, L3 3AF UK; 12 Department of Primary and Community Care, Radboud University Medical Center, Geert Grooteplein Zuid 10, Nijmegen, 6525 GA The Netherlands

**Keywords:** Cardiac rehabilitation, e-Health, Prevention, Sedentary lifestyle, Physical activity, Cardiovascular disease

## Abstract

**Background:**

High sedentary times (ST) is highly prevalent in patients with coronary artery disease (CAD), highlighting the need for behavioural change interventions that effectively reduce ST. We examined the immediate and medium-term effect of the SIT LESS intervention on changes in ST among CAD patients enrolled in cardiac rehabilitation (CR).

**Methods:**

CAD patients participating in CR at 2 regional hospitals were included in this randomized controlled trial (1:1, stratified for gender and hospital). The control group received CR, whereas SIT LESS participants additionally received a 12-week hybrid behaviour change intervention. The primary outcome was the change in accelerometer-derived ST from pre-CR to post-CR and 3 months post-CR. Secondary outcomes included changes in ST and physical activity characteristics, subjective outcomes, and cardiovascular risk factors. A baseline constrained linear mixed-model was used.

**Results:**

Participants (23% female; SIT LESS: *n* = 108, control: *n* = 104) were 63 ± 10 years. Greater ST reductions were found for SIT LESS compared to control post-CR (-1.7 (95% confidence interval (CI): -2.0; -1.4) *versus* − 1.1 (95% CI: -1.4; -0.8) h/day, p_interaction_=0.009), but not at 3 months post-CR (p_interaction_=0.61). Besides, larger light-intensity physical activity (LIPA) increases were found for SIT LESS compared to control post-CR (+ 1.4 (95% CI: +1.2; +1.6) *versus* + 1.0 (95% CI: +0.8; +1.3) h/day, p_interaction_=0.020). Changes in other secondary outcomes did not differ among groups.

**Conclusion:**

SIT LESS transiently reduced ST and increased LIPA, but group differences were no longer significant 3 months post-CR. These findings highlight the challenge to induce sustainable behaviour changes in CAD patients without any continued support.

**Trial registration:**

Netherlands Trial Register: NL9263. Registration Date: 24 February 2021.

**Supplementary Information:**

The online version contains supplementary material available at 10.1186/s12966-024-01642-2.

## Background

Emerging evidence indicates that a daily sedentary time (ST) exceeding the upper limit of normal (i.e. 9.5 h/day) is associated with an increased risk of cardiovascular disease morbidity and mortality in the general population, even after accounting for traditional risk factors [1–3]. A sedentary lifestyle is highly prevalent among patients with coronary artery disease (CAD), as evidenced by substantially higher ST compared to the general population (10.4 *versus* 9.4 h/day) [4, 5]. Epidemiological studies have shown that replacement of ST with light- and moderate-to-vigorous intensity physical activity (LIPA and MVPA) could improve cardiometabolic risk factors [6, 7] and reduce the risk for all-cause and cardiovascular-related mortality [4, 8].

Exercise-based cardiac rehabilitation (CR) programs aim to enhance habitual physical activity (PA) of CAD patients [9], but do not specifically target ST. Consequently, reductions in ST are typically small directly post-CR (0.2–0.4 h/day) [5, 10, 11] and 2 months post-CR (-0.3 h/day) [12], whereas absolute ST remains high after CR completion [13, 14]. Adding a sedentary behaviour change program to current CR seems promising as these programs can effectively induce short-term reductions in ST [15, 16]. Maintaining a reduced ST during follow-up is essential, but studies reporting follow-up measurements are scarce [17].

Therefore, we examined the medium-term effects of a 12-week behaviour change intervention (i.e. SIT LESS) *versus* usual care on changes in objectively measured ST in CAD patients participating in CR. Secondary outcomes included changes in sedentary behaviour characteristics, PA characteristics, quality of life, patients’ competence for self-management, physical function, cardiovascular risk factors, and event-free survival. We hypothesized that SIT LESS would result in a greater decrease in ST compared to usual care.

## Methods

### Setting and population

A parallel-group randomized controlled trial was conducted in order to determine the effectiveness of SIT LESS: a 12-week, hybrid and personalized behaviour change intervention in addition to CR, with a primary focus on reducing and interrupting ST in CAD patients (Netherlands Trial Register: NL9263). The rationale and design of the SIT LESS trial has previously been described in detail including the sample size calculation [18]. There were no changes to the study protocol after trial commencement. Patients from Bernhoven Hospital (Uden, The Netherlands) and Rijnstate Hospital (Arnhem, The Netherlands) were included in this trial if they were aged ≥ 18 years, were referred to CR because of (in)stable angina, myocardial infarction, and/or after coronary revascularization. Furthermore, they had to be able to understand and perform study-related procedures. Exclusion criteria were heart failure with a New York Heart Association class III or IV, physically unable to stand or walk, expected coronary artery bypass grafting (CABG) within eight weeks after inclusion, and concurrent participation in another interventional trial targeting PA [18]. The SIT LESS trial was approved by the Medical Ethics Committee of the Radboud university medical centre (NL72604.091.20), and all participants gave written informed consent. The CONSORT checklist is available in Supplementary Table 1 [19]. The TiDieR Checklist is available in Supplementary Document 1 [20].

### Randomization and masking

Directly after enrolment, the researcher randomly allocated (1:1) the participant into the SIT LESS or control group with allocation concealment in random block sizes ranging from four to six patients, using a computerized algorithm (Castor Electronic Data Capture 2021, Ciwit B.V., Amsterdam, The Netherlands). Randomization was stratified by gender and hospital to ensure balance of the treatment arms. All participants who withdrew within the first two weeks after inclusion were replaced to ensure sufficient power to assess our primary outcome. Nurse specialists and patients were not blinded to the treatment allocation. Primary outcome assessment was blinded for the research team as an automatized script was used based on a unique participant identification number, independent from the randomization procedure.

### Usual care

All participants received usual care, consisting of a ~ 12 week comprehensive CR program. One to three regular, individual consultations were scheduled with the nurse specialist focusing on lifestyle, medication, and psychosocial wellbeing. Participants were offered an outpatient exercise program, consisting of ~ 12 supervised one-hour exercise training sessions across six weeks.

### SIT LESS intervention

Patients in the SIT LESS group received the 12-week SIT LESS intervention alongside usual care CR. SIT LESS was co-created with patients and nurse specialists using the intervention mapping adaptation framework [21] as described elsewhere [18, 22]. Patients received three face-to-face consultations (week 1, 6 and 12, Supplementary Fig. 1) for SIT LESS coaching during which patient education, goal-setting and motivational interviewing with coping planning were addressed. Additionally, patients received a pocket-worn activity tracker connected to a smartphone application (RISE, Appbakkers B.V., Zwolle, The Netherlands). The activity tracker provided vibrotactile feedback after a prolonged (≥ 30 min) sedentary bout and the smartphone application enabled patients and nurse specialists to register personal goals, and review daily ST [23]. Based on the activity tracker-derived ST reports, patients were contacted by telephone for supportive coaching throughout the intervention period.

### Outcomes

The pre-specified primary outcome was the change in objectively measured ST, expressed in h/day, from pre-CR to post-CR and 3 months post-CR [18]. Secondary outcomes included changes in ST characteristics (prolonged sedentary bouts, prevalence of ST ≥ 9.5 h/day), time spent in LIPA and MVPA, step count, quality of life, patients’ competencies for self-management, physical function, cardiovascular risk factors, and incidence of adverse events. Other outcome measures included number of CR consultations attended, number of supervised exercise sessions, and the adherence to the SIT LESS intervention.

### Measurements

#### Accelerometery

ST and PA were assessed using ActivPAL (ActivPAL3^TM^micro, PAL Technologies Ltd., Glasgow, United Kingdom) [24], a small device (25 × 45 × 5 mm), attached to the patient’s thigh using hypoallergenic tape and sealed for waterproof protection. ActivPAL combines a tri-axial accelerometer with an inclinometer which can accurately distinguish between sitting, standing and walking [24]. Patients were instructed to wear the ActivPAL 24 h/day for 8 consecutive days and to fill in a sleep diary. Raw ActivPAL data were analysed by a modified version of the script of Winkler et al [25]. Total ST (Metabolic Equivalent of Task score (METs) ≤ 1.5 while awake in a sitting, lying or reclining posture) [26] was expressed in h/day and the number of prolonged (≥ 30 min) sedentary bouts was calculated. Daily ST was dichotomized using 9.5 h/day as cut-off as it was previously shown that exceeding this limit was associated with an increased risk of morbidity and mortality [1–3]. LIPA (METs < 3) and MVPA (METs ≥ 3) were expressed in h/day, whereas step count was expressed as steps/day. Accelerometery data were gathered pre-CR, post-CR and 3 months post-CR.

For patients randomized to the SIT LESS intervention, we calculated the number of valid wear days of the activity tracker (≥ 10 h/day) to assess the adherence by dividing the number of valid wear days by the total number of days of the intervention period.

### Quality of life and patients’ competence for self-management

Health-related quality of life was assessed using the validated HeartQoL questionnaire (range from 0 (poor) to 3 (better)) [27] at pre-CAD (retrospectively), post-CR, 3 months post-CR, and 9 months post-CR. Global and sub scores for physical and emotional HeartQoL were accordingly computed [27]. Patients’ competencies for self-management of their health were assessed using the validated Dutch 13-Item Patient Activation Measure (PAM-13, scores range from 0 (poor) to 100 (better)) [28] at pre-CAD (retrospectively), post-CR, and 3 months post-CR.

### Physical function

Handgrip strength was assessed with a hydraulic, analogue hand size adjusted dynamometer (Jamar, Jackson, Michigan, USA). Three measurements were conducted for each arm with ± 30 s rest between measurements. Handgrip strength was defined as the maximal value (kg) of the 3 measurements for each arm. The Timed Up and Go was assessed by observing the time while a patient rises from a chair, walks 3 m, turns, walks back and sits down again, as previously described [18, 29]. Physical function was assessed pre-CR and 3 months post-CR.

### Cardiovascular risk factors and adverse events

Patient characteristics, medical history, characteristics of the index hospitalization, and CR characteristics (i.e. number of CR consultations attended; and number of supervised exercise sessions) were derived from electronic patient files (EPF). In addition, the incidence of adverse events, defined as all-cause mortality and major adverse cardiovascular events (MACE; cardiovascular-related rehospitalization, recurrence of acute coronary events, and cardiovascular-related mortality) occurring in the period between the start of CR and the continuing two years, were extracted from the EPF. Socio-economic status was assessed by a telephone interview. Blood samples were obtained pre- and post-CR for assessment of: low-density lipoprotein (LDL)-cholesterol, high-density lipoprotein (HDL)-cholesterol, total cholesterol, triglycerides, haemoglobin, leucocytes, and thrombocytes.

### Statistical analysis

All statistical tests were performed using R version 4.2.1 with packages “lme4”, “survminer” and “survival”. The package ‘LMMstar” was used for visualization purposes. All tests were two-sided, confidence intervals were at the 95% level (95% CI) and P-values < 0.05 were considered statistically significant. Continuous normally distributed data were presented as mean ± standard deviation (SD), continuous not-normally distributed data as median [interquartile range] (IQR) and categorial variables as number (%). All data were visually inspected for normality and the Shapiro-Wilk test was performed.

Primary and secondary outcome analysis were performed on an intention-to-treat basis using a constrained linear mixed-model analysis as baseline imbalances between treatment arms were previously observed [22] and are due to chance, not to bias. Such imbalance can be neutralized in randomized controlled trials using this statistical approach [30], while taking missing data into account (see for more details about the model Supplementary Document 2). The effects of time and time*group interactions were included in the model. The proportion of patients with ST ≥ 9.5 h/day was compared between the SIT LESS and control group at post-CR and 3 months post-CR by constrained logistic mixed-model analysis. Finally, the association between group and adverse events between the start of CR and 2-year of follow-up was assessed using a Kaplan-Meier curve and log-rank test. The hazard ratio (HR) with 95% CI was estimated with a Cox proportional hazard model.

## Results

### Patient characteristics

**Fig. 1:**
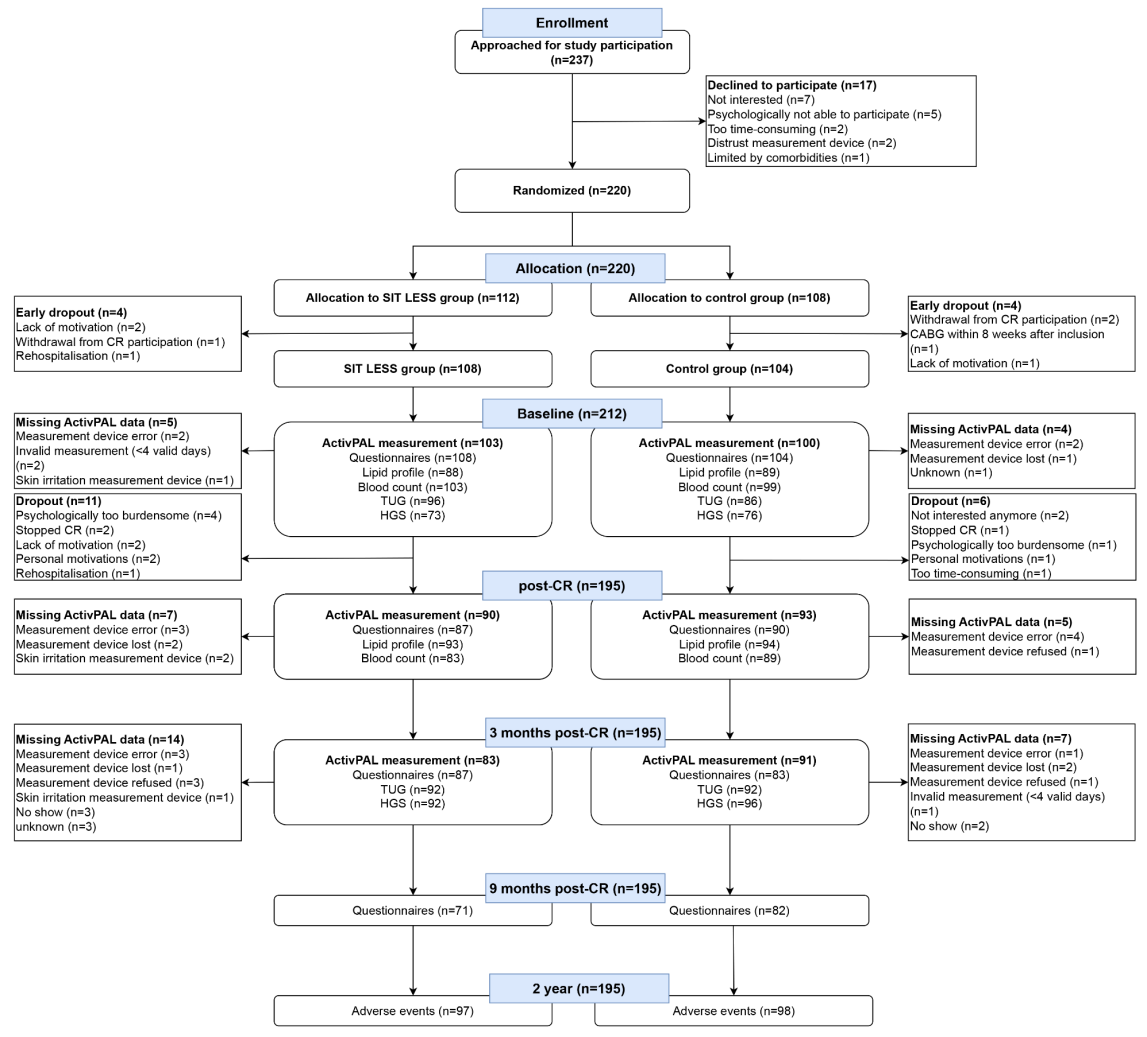
CONSORT flowchart of the SIT LESS randomized controlled trial. In total 237 patients were approached for participation, of which 220 were randomized to either the SIT LESS group or the control group. Eight patients dropped out prior to cardiac rehabilitation (CR) initiation, leaving 108 patients in the SIT LESS group and 104 in the control group. CABG: coronary artery bypass grafting; CR: cardiac rehabilitation; HGS: Handgrip strength; TUG: Timed Up and Go

237 patients were approached for study participation between 30 March and 23 December 2021, of which 212 (89%; SIT LESS: *n* = 108, control: *n* = 104) were included for analysis(Fig. 1). Recruitment was equally distributed between hospitals (*n* = 107 *versusn* = 105). Participants were 63 ± 10 years old, 48 (23%) were female, and index diagnosis was primarily NSTEMI (48%) or STEMI (30%)(Table 1, Supplementary Table 2). There were eleven (10%) drop-outs in the SIT LESS group and six (6%) in the control group(Fig. 1).

**Table 1:** Baseline characteristics of the study cohort

		Total population (*n* = 212)	Missing values (*n* (%))	SIT LESS group (*n* = 108)	Control group (*n* = 104)
**Patient characteristics**					
**Age (years)**		63 (± 10)	0 (0%)	63 (± 10)	64 (± 10)
**Gender (female)**		48 (23%)	0 (0%)	25 (23%)	23 (22%)
**Body mass index (kg/m**^2^)		27.1 [24.6–30.2]	0 (0%)	27.1 [24.6–30.1]	27.2 [24.6–30.8]
**Education level**			20 (9%)		
	Low (n (%))	46 (24%)		19 (20%)	27 (28%)
	Middle (n (%))	77 (40%)		38 (40%)	39 (30%)
	High (n (%))	69 (36%)		38 (40%)	31 (32%)
**Living together/married**		152 (79%)	20 (9%)	77 (81%)	75 (77%)
**Working status**			20 (9%)		
	Employed	98 (51%)		53 (56%)	45 (46%)
	Unemployed	94 (49%)		42 (44%)	52 (54%)
**Living environment**			19 (9%)		
	Transition (n (%))	89 (46%)		43 (45%)	46 (47%)
	Urban (n (%))	54 (28%)		23 (24%)	31 (32%)
	Rural (n (%))	50 (26%)		29 (31%)	21 (21%)
**Regular step count tracking by smartwatch or smartphone (n (%))**		71 (34%)	0 (0%)	33 (31%)	38 (37%)
**Lifestyle factors**					
**Alcohol use (n (%))**		168 (79%)	0 (0%)	87 (81%)	81 (78%)
	Current drinker (n (%))	146 (87%)		75 (86%)	71 (88%)
	Units/week (n)	5 [2–10]		5 [3–9]	5 [2–14]
**Smoking (n (%))**		150 (71%)	0 (0%)	73 (68%)	77 (74%)
	Current smoker (n (%))	41 (27%)		19 (26%)	22 (28%)
	Packyears (n)	23 [10–36]		23 [10–37]	22 [10–36]
**Medical history**					
**Comorbidities**			0 (0%)		
	Hypertension (n (%))	85 (40%)		41 (38%)	44 (42%)
	Dyslipidaemia (n (%))	66 (31%)		34 (32%)	32 (31%)
	Diabetes mellitus (n (%))	36 (17%)		17 (16%)	19 (18%)
	Prior myocardial infarction (n (%))	33 (16%)		18 (17%)	15 (14%)
	Prior PCI (n (%))	26 (12%)		17 (16%)	9 (9%)
	Prior CABG (n (%))	9 (4%)		7 (6%)	2 (2%)
	Atrial fibrillation (n (%))	18 (9%)		7 (7%)	11 (11%)
	Heart failure with reduced ejection fraction (n (%))	17 (8%)		10 (9%)	7 (7%)
	Peripheral artery disease (n (%))	17 (8%)		8 (7%)	9 (9%)
	Heart valve disease (n (%))	15 (7%)		12 (11%)	3 (3%)
	Depression (n (%))	12 (6%)		7 (7%)	5 (5%)
	Cancer (diagnosed in the past 5 years) (n (%))	12 (6%)		8 (7%)	4 (4%)
	Rheumatoid arthritis (n (%))	8 (4%)		4 (4%)	4 (4%)
	COPD (n (%))	8 (4%)		3 (3%)	5 (5%)
	CVA (n (%))	8 (4%)		3 (3%)	5 (5%)
	TIA (n (%))	7 (3%)		3 (3%)	4 (4%)
	Chronic renal failure (eGFR < 30 ml/min/1.73m^2^ or dialysis) (n (%))	7 (3%)		6 (6%)	1 (1%)
**CAD diagnosis at hospitalization**					
**Out of hospital cardiac arrest (n (%))**		7 (3%)	0 (0%)	4 (4%)	3 (3%)
**Index diagnosis**			0 (0%)		
	Non-ST-elevation myocardial infarction (n (%))	102 (48%)		57 (53%)	45 (43%)
	ST-elevation myocardial infarction (n (%))	64 (30%)		30 (28%)	34 (33%)
	Stable angina pectoris (n (%))	30 (14%)		14 (13%)	16 (15%)
	Unstable angina pectoris (n (%))	16 (8%)		7 (7%)	9 (9%)
**Treatment**			0 (0%)		
	PCI (n (%))	126 (59%)		62 (57%)	64 (62%)
	CABG (n (%))	56 (26%)		28 (26%)	28 (27%)
	Conservative (optimal medical treatment only) (n (%))	30 (14%)		18 (17%)	12 (12%)
**Laboratory values**					
	Peak hs-cTnT (ng/L)	1977 [380-19151]	22 (21%)	2083 [437-24275]	1496 [352-16206]
	Peak hs-cTnI (ng/L)	5155 [500-23984]	10 (10%)	3668 [291-25000]	5554 [655-22276]
	Peak CK (U/L)	199 [104–508]	34 (16%)	236 [113–526]	180 [99–475]
	Total cholesterol (mmol/L)	5.0 (± 1.4)	36 (17%)	4.9 (± 1.2)	5.1 (± 1.5)
	LDL-cholesterol (mmol/L)	3.0 (± 1.2)	37 (18%)	2.9 (± 1.1)	3.1 (± 1.3)
	HDL-cholesterol (mmol/L)	1.1 (± 0.3)	36 (17%)	1.2 (± 0.4)	1.1 (± 0.3)
	Triglycerides (mmol/L)	1.4 [1.0-2.2]	36 (17%)	1.4 [1.0–2.0]	1.5 [1.0-2.5]

Data are presented as n (%) for categorical variables and as mean (± standard deviation) for normal distributed continuous data or median [interquartile range] for non-normal distributed continuous variables

CABG: coronary artery bypass grafting; COPD: chronic obstructive pulmonary disease; CK: creatine kinase; CVA: cerebrovascular accident; HDL: High-density lipoprotein; hs-cTnI: high-sensitive cardiac Troponin-I; hs-cTnT: high-sensitive cardiac Troponin-T; LDL: low-density lipoprotein; PCI: Percutaneous Coronary Intervention; TIA: Transient ischemic attack

The CR completion rate in the total cohort was 88% and 90% of patients in the SIT LESS group completed all face-to-face SIT LESS coaching consultations during an intervention period of 89 ± 13 days. During this period, median number of telephone coaching sessions was 7 [6–8] and the adherence to the use of the activity tracker across the entire intervention period was 84 [72–94]%. (Table 2).

**Table 2:** Adherence to cardiac rehabilitation program and SIT LESS intervention

			Total population (*n* = 212)	Missing values (*n* (%))	SIT LESS group (*n* = 108)	Control group (*n* = 104)
**Contemporary CR program**						
Intake consult CR (n (%))			211 (100%)	0 (0%)	107 (99%)	104 (100%)
Exit consult CR (n (%))			197 (96%)	7 (3%)	98 (95%)	99 (97%)
Exercise training at CR (yes) (n (%))			171 (88%)	17 (8%)	85 (88%)	86 (88%)
	Supervised exercise sessions (n)		11 [9–11]	0 (0%)	11 [9–11]	11 [9–12]
**SIT LESS**						
	Intake consult SIT LESS (n (%))				107 (99%)	
	Interim consult SIT LESS (n (%))				101 (94%)	
	Exit consult SIT LESS (n (%))				97 (90%)	
	SIT LESS telephone coaching sessions total intervention period (n (%))				7 [6–8]	
	Duration intervention period (days)*				89 (± 13)	
	Number of days with ≥ 10 h wear time of activity tracker (days)				71 (± 19)	
	Adherence to activity tracker (%)				84 [72–94]	
	Premature discontinuation activity tracker use (n (%))				11 (11%)	
		Before interim consult (n (%))			3 (3%)	
		After interim consult (n (%))			8 (8%)	

Data are presented as n (%) for categorical variables and as mean (± standard deviation) for normal distributed continuous data or median [interquartile range] for non-normal distributed continuous variables. *patients who did not complete CR - SIT LESS coaching were excluded

CR: Cardiac rehabilitation

Study-related adverse events included skin irritation caused by the attachment of the ActivPAL (*n* = 2). The incidence of adverse events was assessed at 6 December 2023 for the analytical cohort.

### Sedentary behaviour

**Fig. 2:**
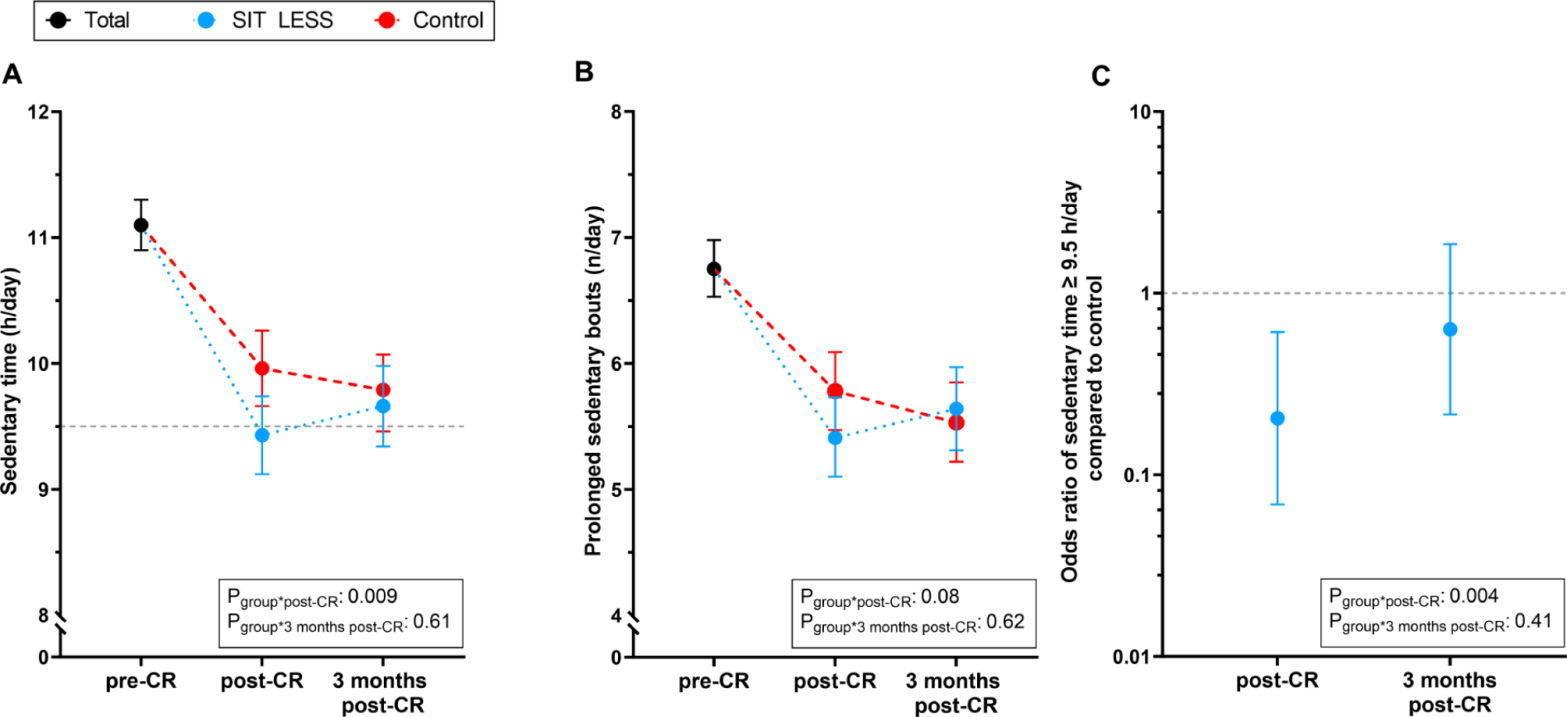
Constrained linear (**A**,** B**) and logistic (**C**) mixed model sedentary behaviour outcomes. The total group (black) pre-cardiac rehabilitation (CR; SIT LESS *n* = 103; control: *n* = 100), and for the SIT LESS (blue) and control (red) group immediately post-CR (SIT LESS: *n* = 90; control: *n* = 93) and 3 months post-CR (SIT LESS: *n* = 83; control: *n* = 91) are depicted. (**A**) Sedentary time in hours per day with the dashed line representing the upper-limit of normal (9.5 h per day). (**B**) Daily number of prolonged sedentary time bouts (≥ 30 min) per day. Data are plotted as mean with 95% confidence intervals. (**C**) Odds ratio of a sedentary time above the upper-limit of normal (≥ 9.5 h/day) for the SIT LESS compared to the control group. P-values depict between group differences (interaction term), with the values post-CR and 3 months post-CR compared to pre-CR

Greater decreases in ST were found for SIT LESS compared to control at post-CR (-1.7 (95% CI: -2.0; -1.4) *versus* − 1.1 (95% CI: -1.4; -0.8) h/day, p_interaction_=0.009), but these differences were no longer significant at 3 months post-CR (-1.4 (95% CI: -1.8; -1.1) *versus* − 1.3 (95% CI: -1.6; -1.0) h/day, p_interaction_=0.61, Fig. 2A, Supplementary Table 3). Similarly, the prevalence of ST ≥ 9.5 h/day was lower in the SIT LESS compared to the control group (Odds ratio (OR): 0.2 (95% CI: 0.1; 0.6)) directly post-CR, but the difference was no longer significant at 3 months post-CR (OR: 0.6 (95% CI: 0.2; 1.9), Fig. 2C). Changes in the number of prolonged sedentary bouts did not differ between the SIT LESS and control group at post-CR (-1.3 (95% CI: -1.7; -1.0) *versus* − 1.0 (95% CI: -1.3; -0.7) bouts/day, p_interaction_=0.08) and at 3 months post-CR (-1.1 (95% CI: -1.4; -0.8) *versus* − 1.2 (95% CI: -1.5; -0.9) bouts/day, p_interaction_=0.62, Fig. 2B).

### Physical activity

**Fig. 3:**
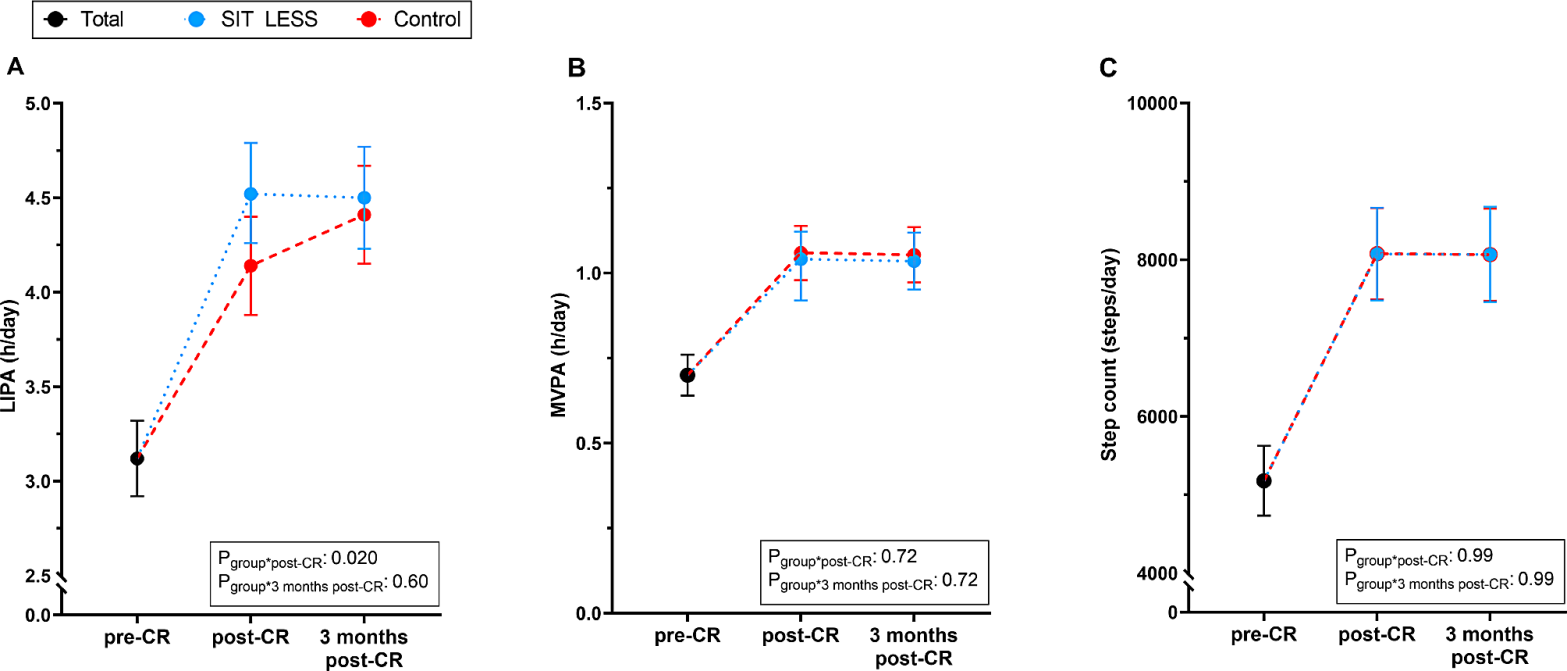
Constrained mixed model physical activity outcomes. The total group (black) pre-cardiac rehabilitation (CR; SIT LESS *n* = 103; control: *n* = 100), and for the SIT LESS (blue) and control (red) group immediately post-CR (SIT LESS: *n* = 90; control: *n* = 93) and 3 months post-CR (SIT LESS: *n* = 83; control: *n* = 91) are depicted. (**A**) Time spent in light-intensity physical activity (LIPA). (**B**) Time spent in moderate-to-vigorous intensity physical activity (MVPA). (**C**) Daily number of steps. Values are given per day and as mean and 95% confidence interval

Larger increases in LIPA were found for SIT LESS compared to control at post-CR (+ 1.4 (95% CI: +1.2; +1.6) *versus* + 1.0 (95% CI: +0.8; +1.3) h/day, p_interaction_=0.020), but these differences were no longer significant at 3 months post-CR (+ 1.4 (95% CI: +1.1; +1.6) *versus* + 1.3 (95% CI: +1.1; +1.5) h/day, p_interaction_=0.60, Fig. 3A). Changes in MVPA did not differ among groups at post-CR (+ 0.3 (95% CI: +0.3; +0.4) *versus* + 0.4 (95% CI: +0.3; +0.4) h/day, p_interaction_=0.72) and at 3 months post-CR (+ 0.3 (95% CI: +0.3; +0.4) *versus* + 0.4 (95% CI: +0.3; +0.4) h/day, p_interaction_=0.72, Fig. 3B). Similarly, changes in step count did not differ between groups at post-CR (+ 2,889 (95% CI: +2,327; +3,450) *versus* + 2,899 (95% CI: +2,365; +3,433) steps/day, p_interaction_=0.99) and at 3 months post-CR (+ 2,894 (95% CI: +2,350; +3,438) *versus* + 2,886 (95% CI: +2,348; +3,425) steps/day, p_interaction_=0.99, Fig. 3C).

### Secondary outcomes

**Fig. 4:**
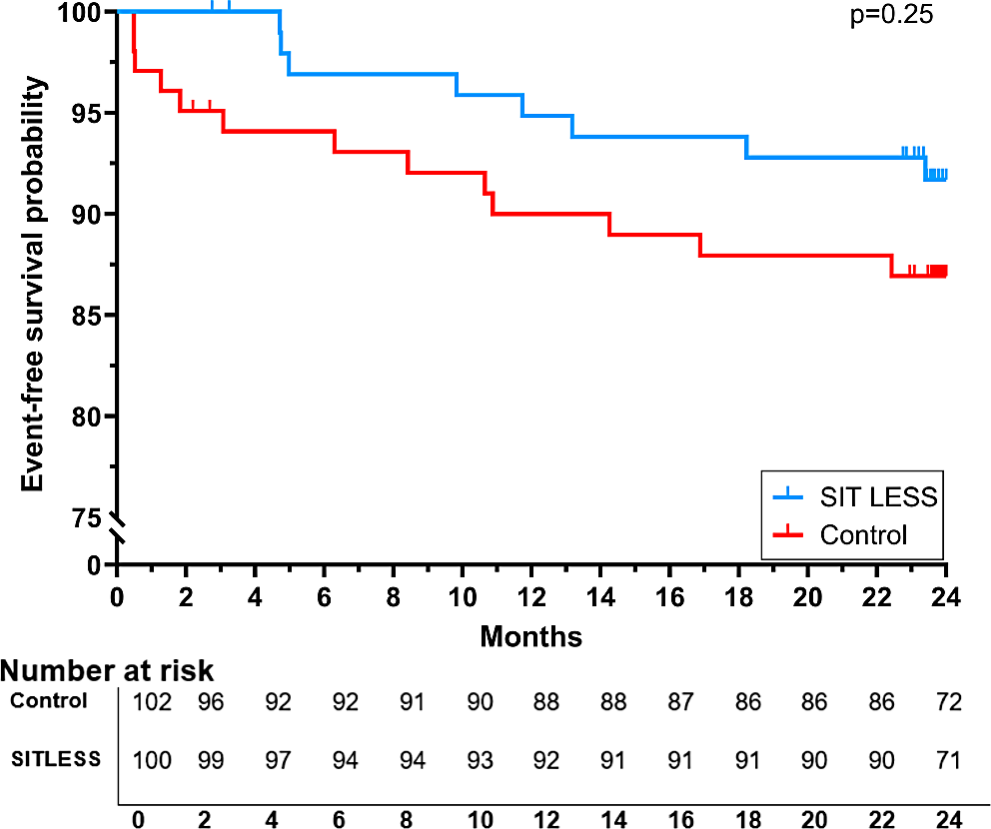
Kaplan-Meier curve of the time to events for the SIT LESS (blue) and control (red) group. Events were defined as all-cause mortality and major adverse cardiovascular events (MACE; cardiovascular-related hospitalization and recurrence of acute coronary events). Adverse events occurred in 21 out of 212 participants (10%) between the start of cardiac rehabilitation and 2 years of follow-up. Participants in the SIT LESS group did not have a different event free survival (Hazard ratio: 0.60, 95% CI: 0.25; 1.44, *p* = 0.25) compared to participants in the control group

Changes in quality of life, patients’ competence for self-management and physical functioning did not differ between the SIT LESS and control group (Supplementary Fig. 2 and Supplementary Fig. 3). Changes in HDL-cholesterol differed across groups, with no change in the SIT LESS group versus small improvements in the control group at post-CR (-0.1 (95% CI: -0.2; -0.1) mmol/l, p_interaction_<0.001). Changes in total cholesterol (p_interaction_=0.43), LDL-cholesterol (p_interaction_=0.63, Supplementary Fig. 4) and other cardiovascular risk factors were not different between the groups (Supplementary Fig. 4). Adverse events occurred in 21 participants (10%) during 2-years of follow-up (Fig. 4). The risk for adverse events was not different between the SIT LESS compared to the control group (HR: 0.60 (95% CI: 0.25; 1.44)).

## Discussion

This randomized controlled trial examined the medium-term effects of the SIT LESS intervention in CAD patients participating in CR. We found that SIT LESS induced a greater reduction in ST, lower prevalence of ST ≥ 9.5 h/day and a larger increase in LIPA compared to usual care when assessed directly post-CR, but all of these beneficial behaviour changes were no longer significant at 3 months post-CR. Changes in quality of life, patients’ competence for self-management, physical functioning, cardiovascular risk factors and adverse events did not differ among groups. These collective findings show that adding a hybrid and personalized behaviour change intervention to CR can effectively change ST and LIPA of patients with CAD, but does not result in sustainable lifestyle changes after discontinuation of the intervention.

### Sedentary behaviour and physical activity

The SIT LESS group replaced ST for LIPA to a greater extent during CR compared to the control group, highlighting that sedentary behaviour is modifiable by the SIT LESS intervention. The observed improvements in ST and LIPA were larger than expected, as earlier studies showed a CR-induced reduction in ST of merely 0.4 h/day and an increase in LIPA of 0.3 h/day [5, 11]. Replacing ST with LIPA is likely to be clinically relevant, as prospective cohort studies have shown that small improvements in LIPA (+ 35 min/day) already induce a reduction in mortality risk during long-term follow-up [3]. MVPA did not differ between SIT LESS and control, supporting previous findings that ST and MVPA are distinct behaviours that require specific targeting [16]. Taken together, this randomized clinical trial shows the potential of SIT LESS to reduce ST during traditional CR programs.

Differences in ST and LIPA improvements between the SIT LESS and control group disappeared at 3 months post-CR. It is known that maintaining a healthy lifestyle, encompassing PA and risk factor management, after CR is challenging [31, 32]. Indeed, previous studies report that sedentary behaviour interventions can effectively induce short-term reductions in ST, but fail to maintain these behaviour adaptations during follow-up [15, 16]. These observations highlight an important challenge in preventive cardiology: developing and implementing interventions supporting sustained changes in PA.

Different behaviour change maintenance theories can explain the loss of effect of SIT LESS on ST and LIPA during medium-term follow-up. First, behaviour tend to be maintained when people are satisfied with the behavioral outcomes or they enjoy engaging in the behavior [33]. While it takes effort to reduce daily ST, no (direct) positive effects of SIT LESS on other secondary outcomes were observed. Communication on the positive long-term health outcomes associated with lower ST levels, such as a better event-free survival, are hypothesized to be insufficient for maintenance of behavior [33]. Second, participants were not able to keep the activity tracker post-CR, which limited their possibilities for self-monitoring, an important aspect to prevent relapsing into prior behavior [33]. Third, the 12-weeks SIT LESS intervention period might have been too short to incorporate sustainable behaviour changes into daily living routines. A previous study demonstrated that PA interventions that lasted ≥ 24 weeks and included follow-up prompts were more likely to achieve maintenance of behavior [17]. These factors should be taken into account while refining the SIT LESS intervention prior to implementation in clinical practice.

### Future perspectives

It is recommended that future (sedentary) behaviour interventions incorporate a maintenance eHealth program following completion of CR, as this strategy can effectively preserve PA levels and improve quality of life [31]. Continued support to allow self-monitoring of behaviour, goal-setting and associated feedback are important components to maintain behaviour changes [31]. For example, remote booster programs may be effective in realizing a physically active lifestyle in CR graduates [34]. Alternatively, adding additional behaviour change elements, can assist to achieve behaviour maintenance [33]. The physical environment can be restructured to invite people to transition to standing more often, for example by limiting the number of chairs in public areas and height-adjustable desks [35]. A supportive social environment can be facilitated by shared goal-setting with dyadic interventions [36]. Such a supportive environment can assist participants to maintain their physically active lifestyle post-intervention.

### Strengths and limitations

Strengths of the study include the medium-term evaluation of this novel SIT LESS intervention in combination with a comprehensive assessment of effects on habitual PA characteristics, physical function and adverse events. Nevertheless, there are also some limitations. First, due to the nature of the intervention, investigators, nurse specialists and patients were not blinded for the treatment. This may have contributed to some contamination bias from the SIT LESS intervention to the control group and consequently, an underestimation of the true difference between SIT LESS and controls. Secondly, functional tests outcomes were not collected post-CR and cardiovascular risk factors were not obtained 3 months post-CR for practical reasons. Therefore, it not possible to assess the effect of SIT LESS on all outcome measures at all time points. Finally, we have not systematically acquired information about (maintenance) motives, changes in self-regulation and/or the environment following study participation. It is unclear to what extent these aspects were related to the maintenance of sedentary behaviour changes, as well as to the main reasons for relapse to old sedentary behaviour habits.

## Conclusions

SIT LESS led to a greater reduction in ST, lower prevalence of ST ≥ 9.5 h/day and a larger increase in LIPA compared to usual care directly post-CR, but the effects of this behaviour change intervention were no longer significant at 3 months post-CR. Furthermore, SIT LESS did not affect quality of life, cardiovascular risk factors or adverse events in the short or medium term. Follow-up studies should evaluate the benefits of enriched behaviour change interventions and/or post-CR eHealth maintenance programs to induce sustainable lifestyle changes with less ST and more LIPA and MVPA in CAD patients.

### Electronic supplementary material

Below is the link to the electronic supplementary material.

**Supplementary Table 1**: CONSORT 2010 checklist.

**Supplementary Table 2**: Complete baseline characteristics of the study cohort.

**Supplementary Table 3**: Descriptive statistics and constrained mixed model analysis outcomes.

**Supplementary Figure 1**: SIT LESS randomized controlled trial flowchart.

**Supplementary Figure 2**: Constrained mixed model of the Heart Quality of Life global (A), Heart Quality of Life Physical subscale (B), Heart Quality of Life Emotional subscale (C), and Patient Activation Measure (D).

**Supplementary Figure 3**: Constrained mixed model of the Timed up and go (TUG) (A) and Handgrip strength (HGS) (B).

**Supplementary Figure 4**; Constrained mixed model of the venous blood samples.

**Supplementary Document 1**: TiDieR checklist.

**Supplementary Document 2**: Supplementary methods.

## Data Availability

Data from the SIT LESS randomized controlled trial are available upon reasonable request via the corresponding author.

## Abbreviations

CABG: Coronary Artery Bypass Graft
CAD: Coronary Artery Disease
CR: Cardiac Rehabilitation
HR: Hazard Ratio
LIPA: Light-Intensity Physical Activity
MVPA: Moderate-to-Vigorous intensity Physical Activity
NSTEMI: Non-ST-Elevated Myocardial Infarction
MACE: Major Adverse Cardiovascular Event
NYHA: New York Heart Association
PA: Physical Activity
SIT LESS: Sedentary Behaviour Intervention as a Personalised Secondary Prevention Strategy
ST: Sedentary Time
STEMI: ST-Elevated Myocardial Infarction
