# Supplementary material for: A cardiac-rehab behaviour intervention to reduce sedentary time in coronary artery disease patients: the SIT LESS randomized controlled trial
Source: Int J Behav Nutr Phys Act. 2024 Aug 19;21:90. doi: 10.1186/s12966-024-01642-2 (PMC11331608; doi:10.1186/s12966-024-01642-2)
Supplement: Supplementary file 1 — Supplementary Table 1: CONSORT 2010 checklist. [file 12966_2024_1642_MOESM1_ESM.pdf]

A cardiac-rehab behaviour intervention to reduce sedentary time in coronary artery disease patients: The SIT LESS Randomized Controlled Trial

Sophie H. Kroesen, MSc[a]; Bram M.A. van Bakel, MD, PhD[a]; Marijn de Bruin, PhD[b]; Arzu Günal, MD[c]; Arko Scheepmaker, MD[c]; Wim R.M. Aengevaeren, MD, PhD[d]; Frank F. Willems, MD, PhD[d]; Roderick Wondergem, PhD[e,f,g]; Martijn F. Pisters, PhD[e,f,g]; Francisco B. Ortega, PhD[h,i,j]; Maria T.E. Hopman, MD, PhD[a]; Dick H.J. Thijssen, PhD[a,k]; Esmée A. Bakker, PhD[a,h,l]; Thijs M.H. Eijsvogels, PhD[a]

Affiliations:

[a] Radboud university medical center, Department of Medical BioSciences, Geert Grooteplein Zuid 10, 6525 GA, Nijmegen, The Netherlands.

[b] Radboud university medical center, Department of IQ healthcare, Geert Grooteplein Zuid 10, 6525 GA, Nijmegen, The Netherlands.

[c] Bernhoven hospital, Department of Cardiology, Nistelrodeseweg 10, 5406 PT, Uden, The Netherlands

[d] Rijnstate hospital, Department of Cardiology, Wagnerlaan 55, 6815 AD, Arnhem, The Netherlands

[e] Utrecht University, University Medical Centre Utrecht Brain Centre, Physical Therapy Science and Sport, Department of Rehabilitation, Universiteitsweg 100, 3584 CG, Utrecht, The Netherlands

[f] Fontys University of Applied Sciences, Department of Health Innovations and Technology, Research Group Empowering Healthy Behaviour, Rachelsmolen 1, 5612 MA, Eindhoven, The Netherlands

[g] Julius Health Care Centres, Centre for Physical Therapy Research and Innovation in Primary Care, Universiteitsweg 100, 3584 CG, Utrecht, the Netherlands

[h] University of Granada, Sport and Health University Research Institute (iMUDS), Department of Physical Education and Sports, Parque Tecnologico de la Salud, Av. del Conocimiento, s/n, 18007, Granada, Spain.

[i] CIBERobn Physiopathology of Obesity and Nutrition, Av. Monforte de Lemos, 3-5. Pabellón 11. Planta 0 28029, Madrid, Spain

[j] University of Jyväskylä, Faculty of Sport and Health Sciences, Keskussairaalantie 4, 40600, Jyväskylä, Finland

[k] Liverpool John Moores University, Research Institute for Sports and Exercise Sciences, Tom Reilly Building, Byrom Street, Liverpool, L3 3AF, United Kingdom

[l] Radboud university medical center, Department of Primary and Community Care, Geert Grooteplein Zuid 10, 6525 GA, Nijmegen, The Netherlands.

Supplementary Table 1. CONSORT 2010 checklist

Section/Topic	Item No	Checklist item	Reported on page No
**Title and abstract**			
	1a	Identification as a randomised trial in the title	1
	1b	Structured summary of trial design, methods, results, and conclusions (for specific guidance see CONSORT for abstracts)	2
**Introduction**			
Background and objectives	2a	Scientific background and explanation of rationale	3
	2b	Specific objectives or hypotheses	3
**Methods**			
Trial design	3a	Description of trial design (such as parallel, factorial) including allocation ratio	4
	3b	Important changes to methods after trial commencement (such as eligibility criteria), with reasons	4
Participants	4a	Eligibility criteria for participants	4
	4b	Settings and locations where the data were collected	4
Interventions	5	The interventions for each group with sufficient details to allow replication, including how and when they were actually administered	5
Outcomes	6a	Completely defined pre-specified primary and secondary outcome measures, including how and when they were assessed	6-7
	6b	Any changes to trial outcomes after the trial commenced, with reasons	4
Sample size	7a	How sample size was determined	4

Section/Topic	Item No	Checklist item	Reported on page No
	7b	When applicable, explanation of any interim analyses and stopping guidelines	NA
Randomisation:			
Sequence generation	8a	Method used to generate the random allocation sequence	4
	8b	Type of randomisation; details of any restriction (such as blocking and block size)	4-5
Allocation concealment mechanism	9	Mechanism used to implement the random allocation sequence (such as sequentially numbered containers), describing any steps taken to conceal the sequence until interventions were assigned	4-5
Implementation	10	Who generated the random allocation sequence, who enrolled participants, and who assigned participants to interventions	4-5
Blinding	11a	If done, who was blinded after assignment to interventions (for example, participants, care providers, those assessing outcomes) and how	4-5
	11b	If relevant, description of the similarity of interventions	NA
Statistical methods	12a	Statistical methods used to compare groups for primary and secondary outcomes	8
	12b	Methods for additional analyses, such as subgroup analyses and adjusted analyses	NA
**Results**			
Participant flow (a diagram is strongly recommended)	13a	For each group, the numbers of participants who were randomly assigned, received intended treatment, and were analysed for the primary outcome	8-9, Figure 1
	13b	For each group, losses and exclusions after randomisation, together with reasons	Figure 1
Recruitment	14a	Dates defining the periods of recruitment and follow-up	8-9
	14b	Why the trial ended or was stopped	NA

Section/Topic	Item No	Checklist item	Reported on page No
Baseline data	15	A table showing baseline demographic and clinical characteristics for each group	Table 1, Supplementary Table 2
Numbers analysed	16	For each group, number of participants (denominator) included in each analysis and whether the analysis was by original assigned groups	Supplementary Table 3
Outcomes and estimation	17a	For each primary and secondary outcome, results for each group, and the estimated effect size and its precision (such as 95% confidence interval)	12-13, Supplementary Table 3
	17b	For binary outcomes, presentation of both absolute and relative effect sizes is recommended	9-10, Table 1, Supplementary Table 2
Ancillary analyses	18	Results of any other analyses performed, including subgroup analyses and adjusted analyses, distinguishing pre-specified from exploratory	NA
Harms	19	All important harms or unintended effects in each group (for specific guidance see CONSORT for harms)	9
**Discussion**			
Limitations	20	Trial limitations, addressing sources of potential bias, imprecision, and, if relevant, multiplicity of analyses	13
Generalisability	21	Generalisability (external validity, applicability) of the trial findings	10-13
Interpretation	22	Interpretation consistent with results, balancing benefits and harms, and considering other relevant evidence	10-13
**Other information**			
Registration	23	Registration number and name of trial registry	2,4
Protocol	24	Where the full trial protocol can be accessed, if available	4

Section/Topic	Item No	Checklist item	Reported on page No
Funding	25	Sources of funding and other support (such as supply of drugs), role of funders	15
