# Supplementary material for: A cardiac-rehab behaviour intervention to reduce sedentary time in coronary artery disease patients: the SIT LESS randomized controlled trial
Source: Int J Behav Nutr Phys Act. 2024 Aug 19;21:90. doi: 10.1186/s12966-024-01642-2 (PMC11331608; doi:10.1186/s12966-024-01642-2)
Supplement: Supplementary file 2 — Supplementary Table 2: Complete baseline characteristics of the study cohort. [file 12966_2024_1642_MOESM2_ESM.pdf]

A cardiac-rehab behaviour intervention to reduce sedentary time in coronary artery disease patients: The SIT LESS Randomized Controlled Trial

Sophie H. Kroesen, MSc[a]; Bram M.A. van Bakel, MD, PhD[a]; Marijn de Bruin, PhD[b]; Arzu Günal, MD[c]; Arko Scheepmaker, MD[c]; Wim R.M. Aengevaeren, MD, PhD[d]; Frank F. Willems, MD, PhD[d]; Roderick Wondergem, PhD[e,f,g]; Martijn F. Pisters, PhD[e,f,g]; Francisco B. Ortega, PhD[h,i,j]; Maria T.E. Hopman, MD, PhD[a]; Dick H.J. Thijssen, PhD[a,k]; Esmée A. Bakker, PhD[a,h,l]; Thijs M.H. Eijsvogels, PhD[a]

Affiliations:

[a] Radboud university medical center, Department of Medical BioSciences, Geert Grooteplein Zuid 10, 6525 GA, Nijmegen, The Netherlands.

[b] Radboud university medical center, Department of IQ healthcare, Geert Grooteplein Zuid 10, 6525 GA, Nijmegen, The Netherlands.

[c] Bernhoven hospital, Department of Cardiology, Nistelrodeseweg 10, 5406 PT, Uden, The Netherlands

[d] Rijnstate hospital, Department of Cardiology, Wagnerlaan 55, 6815 AD, Arnhem, The Netherlands

[e] Utrecht University, University Medical Centre Utrecht Brain Centre, Physical Therapy Science and Sport, Department of Rehabilitation, Universiteitsweg 100, 3584 CG, Utrecht, The Netherlands

[f] Fontys University of Applied Sciences, Department of Health Innovations and Technology, Research Group Empowering Healthy Behaviour, Rachelsmolen 1, 5612 MA, Eindhoven, The Netherlands

[g] Julius Health Care Centres, Centre for Physical Therapy Research and Innovation in Primary Care, Universiteitsweg 100, 3584 CG, Utrecht, the Netherlands

[h] University of Granada, Sport and Health University Research Institute (iMUDS), Department of Physical Education and Sports, Parque Tecnologico de la Salud, Av. del Conocimiento, s/n, 18007, Granada, Spain.

[i] CIBERobn Physiopathology of Obesity and Nutrition, Av. Monforte de Lemos, 3-5. Pabellón 11. Planta 0 28029, Madrid, Spain

[j] University of Jyväskylä, Faculty of Sport and Health Sciences, Keskussairaalantie 4, 40600, Jyväskylä, Finland

[k] Liverpool John Moores University, Research Institute for Sports and Exercise Sciences, Tom Reilly Building, Byrom Street, Liverpool, L3 3AF, United Kingdom

[l] Radboud university medical center, Department of Primary and Community Care, Geert Grooteplein Zuid 10, 6525 GA, Nijmegen, The Netherlands.

Supplementary Table 2. Complete baseline characteristics of the study cohort.

		Total population<br><br>(n=212)	Missing values (n (%))	SIT LESS group<br><br>(n=108)	Control group<br><br>(n=104)
**Patient characteristics**					
Age (years)		63 (±10)	0 (0%)	63 (±10)	64 (±10)
Gender (female)		48 (23%)	0 (0%)	25 (23%)	23 (22%)
Body mass index (kg/m^2)		27.1 [24.6-30.2]	0 (0%)	27.1 [24.6-30.1]	27.2 [24.6-30.8]
Nationality			0 (0%)		
	Dutch (n (%))	201 (95%)		104 (96%)	97 (93%)
	Non-Dutch (n (%))	11 (5%)		4 (4%)	7 (7%)
Education level			20 (9%)		
	Low (n (%))	46 (24%)		19 (20%)	27 (28%)
	Middle (n (%))	77 (40%)		38 (40%)	39 (30%)
	High (n (%))	69 (36%)		38 (40%)	31 (32%)
Living together/married		152 (79%)	20 (9%)	77 (81%)	75 (77%)
Working status					
Employed		98 (51%)	20 (9%)	53 (56%)	45 (46%)
	Mainly sedentary at work (n (%))	35 (36%)		20 (38%)	15 (33%)
	Some light physical activities at work (n (%))	20 (20%)		13 (24%)	7 (16%)
	Light to moderate physical activities at work (n (%))	10 (10%)		4 (8%)	6 (13%)
	Moderate to vigorous physical activities at work (n (%))	33 (34%)		16 (30%)	17 (38%)
Unemployed		94 (49%)	20 (9%)	42 (44%)	52 (54%)
	Retirement (n (%))	80 (85%)		37 (88%)	43 (83%)
	Health problems (n (%))	13 (14%)		4 (10%)	9 (17%)
	Household tasks (n (%))	1 (1%)		1 (2%)	0 (0%)
Monthly household income (€)			37 (17%)		
	< 2000 (n (%))	35 (20%)		19 (22%)	16 (17%)
	2000 – 3999 (n (%))	95 (54%)		44 (52%)	51 (56%)
	4000 – 5999 (n (%))	35 (20%)		18 (21%)	17 (19%)

	≥ 6000 (n (%))	10 (6%)		4 (5%)	6 (8%)
Living environment			19 (9%)		
	Transition (n (%))	89 (46%)		43 (45%)	46 (47%)
	Urban (n (%))	54 (28%)		23 (24%)	31 (32%)
	Rural (n (%))	50 (26%)		29 (31%)	21 (21%)
Regular step count tracking by smartwatch or smartphone (n (%))		71 (34%)	0 (0%)	33 (31%)	38 (37%)
**Lifestyle factors**					
Alcohol use (n (%))		168 (79%)	0 (0%)	87 (81%)	81 (78%)
	Current drinker (n (%))	146 (87%)		75 (86%)	71 (88%)
	Units/week (n)	5 [2-10]		5 [3-9]	5 [2-14]
Smoking (n (%))		150 (71%)	0 (0%)	73 (68%)	77 (74%)
	Current smoker (n (%))	41 (27%)		19 (26%)	22 (28%)
	Packyears (n)	23 [10-36]		23 [10-37]	22 [10-36]
**Medical history**					
Comorbidities			0 (0%)		
	Hypertension (n (%))	85 (40%)		41 (38%)	44 (42%)
	Dyslipidaemia (n (%))	66 (31%)		34 (32%)	32 (31%)
	Diabetes mellitus (n (%))	36 (17%)		17 (16%)	19 (18%)
	Prior myocardial infarction (n (%))	33 (16%)		18 (17%)	15 (14%)
	Prior PCI (n (%))	26 (12%)		17 (16%)	9 (9%)
	Prior CABG (n (%))	9 (4%)		7 (6%)	2 (2%)
	Atrial fibrillation (n (%))	18 (9%)		7 (7%)	11 (11%)
	Heart failure with reduced ejection fraction (n (%))	17 (8%)		10 (9%)	7 (7%)
	Peripheral artery disease (n (%))	17 (8%)		8 (7%)	9 (9%)
	Heart valve disease (n (%))	15 (7%)		12 (11%)	3 (3%)
	Depression (n (%))	12 (6%)		7 (7%)	5 (5%)
	Cancer (diagnosed in the past 5 years) (n (%))	12 (6%)		8 (7%)	4 (4%)
	Rheumatoid arthritis (n (%))	8 (4%)		4 (4%)	4 (4%)
	COPD (n (%))	8 (4%)		3 (3%)	5 (5%)

CVA (n (%))	8 (4%)		3 (3%)	5 (5%)
TIA (n (%))	7 (3%)		3 (3%)	4 (4%)
Chronic renal failure (eGFR<30 ml/min/1.73m^2 or dialysis) (n (%))	7 (3%)		6 (6%)	1 (1%)
**CAD diagnosis at hospitalization**				
Out of hospital cardiac arrest (n (%))	7 (3%)	0 (0%)	4 (4%)	3 (3%)
Index diagnosis		0 (0%)		
Non-ST-elevation myocardial infarction (n (%))	102 (48%)		57 (53%)	45 (43%)
ST-elevation myocardial infarction (n (%))	64 (30%)		30 (28%)	34 (33%)
Stable angina pectoris (n (%))	30 (14%)		14 (13%)	16 (15%)
Unstable angina pectoris (n (%))	16 (8%)		7 (7%)	9 (9%)
Coronary angiography findings		0 (0%)		
1-vessel disease (n (%))	113 (53%)		60 (56%)	53 (51%)
2-vessel disease (n (%))	49 (23%)		22 (20%)	27 (26%)
3-vessel disease (n (%))	41 (19%)		20 (19%)	21 (20%)
No significant stenosis (n (%))	9 (4%)		6 (6%)	3 (3%)
Treatment		0 (0%)		
PCI (n (%))	126 (59%)		62 (57%)	64 (62%)
CABG (n (%))	56 (26%)		28 (26%)	28 (27%)
Conservative (optimal medical treatment only) (n (%))	30 (14%)		18 (17%)	12 (12%)
Laboratory values				
Peak hs-cTnT (ng/L)	1977 [380-19151]	22 (21%)	2083 [437-24275]	1496 [352-16206]
Peak hs-cTnI (ng/L)	5155 [500-23984]	10 (10%)	3668 [291-25000]	5554 [655-22276]
Peak CK (U/L)	199 [104-508]	34 (16%)	236 [113-526]	180 [99–475]
Total cholesterol (mmol/L)	5.0 (±1.4)	36 (17%)	4.9 (±1.2)	5.1 (±1.5)
LDL-cholesterol (mmol/L)	3.0 (±1.2)	37 (18%)	2.9 (±1.1)	3.1 (±1.3)
HDL-cholesterol (mmol/L)	1.1 (±0.3)	36 (17%)	1.2 (±0.4)	1.1 (±0.3)
Triglycerides (mmol/L)	1.4 [1.0-2.2]	36 (17%)	1.4 [1.0-2.0]	1.5 [1.0-2.5]
In-hospital complications (n (%))	17 (8%)	0 (0%)	8 (8%)	9 (9%)

Complicated PCI (n (%))	6 (3%)		3 (3%)	3 (3%)
Complicated CABG (n (%))	5 (2%)		2 (2%)	3 (3%)
Target vessel revascularization (n (%))	2 (1%)		1 (1%)	1 (1%)
Ischemic CVA (n (%))	2 (1%)		1 (1%)	1 (1%)
Major bleeding (n (%))	1 (0.5%)		0 (0%)	1 (1%)
In hospital cardiac arrest (n (%))	1 (0.5%)		1 (1%)	0 (0%)
Duration of hospitalization (days)	5 [3-9]	0 (0%)	5 [3-9]	5 [3-10]
**Cardiovascular medication pre-CR**				
Acetylsalicylic acid (n (%))	170 (85%)	11 (5%)	86 (87%)	84 (82%)
ACE-inhibitor/Angiotensin receptor blocker (n (%))	145 (72%)	11 (5%)	77 (78%)	68 (67%)
Beta-blocker (n (%))	166 (83%)	11 (5%)	80 (81%)	86 (84%)
Platelet aggregation inhibitor	165 (82%)	11 (5%)	84 (85%)	81 (79%)
Statins (n (%))	188 (94%)	13 (5%)	93 (94%)	95 (93%)

Data are presented as n (%) for categorical variables and as mean (± standard deviation) for normal distributed continuous data or median [interquartile range] for non-normal distributed continuous variables.

ACE: Angiotensin-converting enzyme; CABG: coronary artery bypass grafting; COPD: chronic obstructive pulmonary disease; CK: creatine kinase; CVA: cerebrovascular accident; HDL: High-density lipoprotein; hs-cTnI: high-sensitive cardiac Troponin-I; hs-cTnT: high-sensitive cardiac Troponin-T; LDL: low-density lipoprotein; PCI: Percutaneous Coronary Intervention; TIA: Transient ischemic attack.
