# Supplementary material for: A cardiac-rehab behaviour intervention to reduce sedentary time in coronary artery disease patients: the SIT LESS randomized controlled trial
Source: Int J Behav Nutr Phys Act. 2024 Aug 19;21:90. doi: 10.1186/s12966-024-01642-2 (PMC11331608; doi:10.1186/s12966-024-01642-2)
Supplement: Supplementary file 3 — Supplementary Table 3: Descriptive statistics and constrained mixed model analysis outcomes. [file 12966_2024_1642_MOESM3_ESM.pdf]

A cardiac-rehab behaviour intervention to reduce sedentary time in coronary artery disease patients: The SIT LESS Randomized Controlled Trial

Sophie H. Kroesen, MSc[a]; Bram M.A. van Bakel, MD, PhD[a]; Marijn de Bruin, PhD[b]; Arzu Günal, MD[c]; Arko Scheepmaker, MD[c]; Wim R.M. Aengevaeren, MD, PhD[d]; Frank F. Willems, MD, PhD[d]; Roderick Wondergem, PhD[e,f,g]; Martijn F. Pisters, PhD[e,f,g]; Francisco B. Ortega, PhD[h,i,j]; Maria T.E. Hopman, MD, PhD[a]; Dick H.J. Thijssen, PhD[a,k]; Esmée A. Bakker, PhD[a,h,l]; Thijs M.H. Eijsvogels, PhD[a]

Affiliations:

[a] Radboud university medical center, Department of Medical BioSciences, Geert Grooteplein Zuid 10, 6525 GA, Nijmegen, The Netherlands.

[b] Radboud university medical center, Department of IQ healthcare, Geert Grooteplein Zuid 10, 6525 GA, Nijmegen, The Netherlands.

[c] Bernhoven hospital, Department of Cardiology, Nistelrodeseweg 10, 5406 PT, Uden, The Netherlands

[d] Rijnstate hospital, Department of Cardiology, Wagnerlaan 55, 6815 AD, Arnhem, The Netherlands

[e] Utrecht University, University Medical Centre Utrecht Brain Centre, Physical Therapy Science and Sport, Department of Rehabilitation, Universiteitsweg 100, 3584 CG, Utrecht, The Netherlands

[f] Fontys University of Applied Sciences, Department of Health Innovations and Technology, Research Group Empowering Healthy Behaviour, Rachelsmolen 1, 5612 MA, Eindhoven, The Netherlands

[g] Julius Health Care Centres, Centre for Physical Therapy Research and Innovation in Primary Care, Universiteitsweg 100, 3584 CG, Utrecht, the Netherlands

[h] University of Granada, Sport and Health University Research Institute (iMUDS), Department of Physical Education and Sports, Parque Tecnologico de la Salud, Av. del Conocimiento, s/n, 18007, Granada, Spain.

[i] CIBERobn Physiopathology of Obesity and Nutrition, Av. Monforte de Lemos, 3-5. Pabellón 11. Planta 0 28029, Madrid, Spain

[j] University of Jyväskylä, Faculty of Sport and Health Sciences, Keskussairaalantie 4, 40600, Jyväskylä, Finland

[k] Liverpool John Moores University, Research Institute for Sports and Exercise Sciences, Tom Reilly Building, Byrom Street, Liverpool, L3 3AF, United Kingdom

[l] Radboud university medical center, Department of Primary and Community Care, Geert Grooteplein Zuid 10, 6525 GA, Nijmegen, The Netherlands.

Supplementary Table 3. Descriptive statistics and constrained mixed model analysis outcomes.

	Descriptive statistics<br><br>Total (n=212)	Descriptive statistics<br><br>SIT LESS (n=108)	Descriptive statistics<br><br>Control (n=104)	Constrained mixed model<br><br>Time*group interaction (95% CI)	Constrained mixed model<br><br>Cohen's d (95% CI)	Constrained mixed model<br><br>p-value interaction
**Accelerometry**						
Sedentary time (h/day)						
Pre-CR (n=203)	11.1±1.6	10.9±1.6	11.3±1.6			
Post-CR (n=183)	9.7±1.8	9.2±1.8	10.1±1.6	-0.5 (-0.9; -0.1)	-0.3 (-0.6; -0.1)	**0.009**
3 months post-CR (n=174)	9.7±1.6	9.5±1.6	9.9±1.5	-0.1 (-0.5; 0.3)	-0.1 (-0.3; 0.2)	0.61
Prolonged sedentary bouts (n/day)						
Pre-CR (n=203)	6.8±1.7	6.5±1.8	7.0±1.6			
Post-CR (n=183)	5.8±1.7	5.3±1.8	5.9±1.5	-0.4 (-0.8; 0.0)	-0.2 (-0.5; 0.0)	0.08
3 months post-CR (n=174)	5.6±1.6	5.5±1.6	5.7±1.5	0.1 (-0.3; 0.5)	0.1 (-0.2; 0.3)	0.62
Prevalence of a sedentary time ≥ 9.5 h/day (n, (%))						
Pre-CR (n=203)	168 (83%)	81 (79%)	87 (87%)			
Post-CR (n=183)	110 (60%)	43 (48%)	67 (72%)	0.2 (0.1; 0.6)[a]		**0.004**
3 months post-CR (n=174)	100 (57%)	43 (52%)	57 (63%)	0.6 (0.2; 1.9) [a]		0.41

	Descriptive statistics<br><br>Total (n=212)	Descriptive statistics<br><br>SIT LESS (n=108)	Descriptive statistics<br><br>Control (n=104)	Constrained mixed model<br><br>Time*group interaction (95% CI)	Constrained mixed model<br><br>Cohen's d (95% CI)	Constrained mixed model<br><br>p-value interaction
Light intensity physical activity (h/day)						
Pre-CR (n=203)	3.1±1.4	3.3±1.4	2.9±1.3			
Post-CR (n=183)	4.3±1.6	4.7±1.6	4.0±1.5	0.4 (0.1; 0.7)	0.3 (0.0; 0.5)	**0.020**
3 months post-CR (n=174)	4.4±1.4	4.7±1.4	4.3±1.4	0.1 (-0.2; 0.4)	0.1 (-0.2; 0.3)	0.60
Moderate-to-vigorous intensity physical activity (h/day)						
Pre-CR (n=203)	0.7±0.4	0.8±0.4	0.6±0.4			
Post-CR (n=183)	1.1±0.5	1.1±0.4	1.0±0.5	-0.0 (-0.1; 0.1)	-0.0 (-0.3; 0.2)	0.72
3 months post-CR (n=174)	1.0±0.5	1.1±0.5	1.0±0.5	-0.0 (-0.1; 0.1)	-0.0 (-0.3; 0.2)	0.72
Step count (steps/day *10^3)						
Pre-CR (n=203)	4.6 [3.2 – 7.0]	4.9 [3.4 – 7.2]	4.1 [2.8 – 6.1]			
Post-CR (n=183)	7.7 [6.0 – 9.9]	8.2 [6.9 – 9.8]	6.8 [5.5 – 10.0]	-0.0 (-0.7; 0.7)	0.0 (-0.2; 0.2)	0.99
3 months post-CR (n=174)	7.9 [5.5 – 9.8]	8.0 [5.7 – 10.3]	7.2 [5.3 – 8.8]	-0.0 (-0.7; 0.7)	0.0 (-0.2; 0.2)	0.99

	Descriptive statistics<br><br>Total (n=212)	Descriptive statistics<br><br>SIT LESS (n=108)	Descriptive statistics<br><br>Control (n=104)	Constrained mixed model<br><br>Time*group interaction (95% CI)	Constrained mixed model<br><br>Cohen's d (95% CI)	Constrained mixed model<br><br>p-value interaction
**HeartQoL and Patients' competencies for self-management**						
HeartQoL total						
Pre-CAD diagnosis (n=212)	2.4 [1.8 – 2.8]	2.5 [1.9 – 2.9]	2.4 [1.8 – 2.7]			
Post-CR (n=177)	2.1 [1.6 – 2.6]	2.2 [1.6 – 2.6]	2.1 [1.7 – 2.6]	-0.0 (-0.2; 0.2)	-0.0 (0.3; 0.2)	0.98
3 months post-CR (n=170)	2.2 [1.6 – 2.9]	2.4 [1.8 – 2.9]	2.1 [1.5 – 2.8]	0.0 (-0.2; 0.2)	0.0 (-0.3; 0.3)	0.96
9 months post-CR (n=153)	2.3 [1.6 – 2.9]	2.4 [1.8 – 2.9]	2.2 [1.6 – 2.7]	0.1 (-0.1; 0.3)	0.2 (-0.1; 0.4)	0.24
HeartQoL physical						
Pre-CAD diagnosis (n=212)	2.6 [1.4 – 2.7]	2.6 [1.8 – 2.9]	2.6 [1.8 – 2.9]			
Post-CR(n=177)	2.2 [1.4 – 2.7]	2.4 [1.5 – 2.8]	2.1 [1.4 – 2.7]	0.0 (-0.1; 0.2)	0.1 (-0.2; 0.3)	0.64
3 months post-CR (n=170)	2.2 [1.5 – 2.8]	2.4 [1.7 – 2.8]	2.2 [1.4 – 2.9]	0.1 (-0.1; 0.3)	0.1 (-0.2; 0.3)	0.50
9 months post-CR (n=153)	2.2 [1.4 – 2.8]	2.4 [1.7 – 2.9]	2.0 [1.4 – 2.7]	0.2 (-0.0; 0.4)	0.2 (-0.0; 0.5)	0.10

		Descriptive statistics<br><br>Total (n=212)	Descriptive statistics<br><br>SIT LESS (n=108)	Descriptive statistics<br><br>Control (n=104)	Constrained mixed model<br><br>Time*group interaction (95% CI)	Constrained mixed model<br><br>Cohen's d (95% CI)	Constrained mixed model<br><br>p-value interaction
HeartQoL emotional							
	Pre-CAD diagnosis (n=212)	2.5 [1.5 – 3.0]	2.5 [1.8 – 3.0]	2.3 [1.5 – 3.0]			
	Post-CR (n=177)	2.5 [1.8 – 3.0]	2.5 [1.8 – 3.0]	2.5 [1.6 – 2.9]	-0.1 (-0.3; 0.1)	-0.1 (-0.4; 0.1)	0.29
	3 months post-CR (n=170)	2.5 [1.8 – 3.0]	2.5 [1.8 – 3.0]	2.5 [1.8 – 3.0]	-0.1 (-0.3; 0.1)	-0.2 (-0.4; 0.1)	0.20
	9 months post-CR (n=153)	2.5 [1.8 – 3.0]	2.8 [2.0 – 3.0]	2.5 [1.8 – 3.0]	-0.0 (-0.3; 0.2)	-0.0 (-0.3; 0.2)	0.73
Patients' competencies for self-management							
	Pre-CAD diagnosis (n=212)	58 [53 – 66]	58 [53 – 63]	58 [51 – 68]			
	Post-CR (n=177)	58 [51 – 69]	58 [51 – 53]	56 [53 – 63]	-0 (-3; 3)	-0.0 (-0.3; 0.3)	0.92
	3 months post-CR (n=170)	56 [49 – 63]	56 [51 – 63]	53 [49 – 63]	1 (-2; 5)	0.1 (-0.2; 0.4)	0.35
**Physical function**							
Timed up and go (s)							
	Pre-CR (n=182)	10 [8 – 11]	10 [9 – 11]	10 [8 – 12]			
	3 months post-CR (n=184)	8 [7 – 10]	8 [7 – 10]	9 [7 - 10]	-0 (-1; 0)	-0.1 (-0.3; 0.2)	0.56

		Descriptive statistics<br><br>Total (n=212)	Descriptive statistics<br><br>SIT LESS (n=108)	Descriptive statistics<br><br>Control (n=104)	Constrained mixed model<br><br>Time*group interaction (95% CI)	Constrained mixed model<br><br>Cohen's d (95% CI)	Constrained mixed model<br><br>p-value interaction
Handgrip strength (kg)							
	Pre-CR (n=149)	40 [32 - 50]	39 [32 – 48]	40 [34 – 50]			
	3 months post-CR (n=186)	45 [36 – 53]	45 [36 – 52]	44 [36 – 54]	2 (-1; 4)	0.1 (-0.0; 0.3)	0.14
**Cardiovascular risk factors**							
Total cholesterol (mmol/l)							
	Pre-CR (n=176)	5.0 [3.9 – 5.8]	4.8 [3.9 – 5.8]	5.0 [3.8 – 5.9]			
	Post-CR (n=187)	3.5 [3.2 – 4.1]	3.5 [3.1 – 4.0]	3.7 [3.3 – 4.3]	-0.1 (-0.4; 0.2)	-0.1 (-0.4; 0.2)	0.44
LDL-cholesterol (mmol/l)							
	Pre-CR (n=175)	3.0 [2.1 – 3.7]	3.0 [2.1 – 3.7]	3.0 [2.1 – 3.7]			
	Post-CR (n=187)	1.7 [1.4 – 2.1]	1.7 [1.3 – 2.1]	1.7 [1.4 – 2.3]	-0.1 (-0.4; 0.2)	-0.1 (-0.3; 0.2)	0.63
HDL-cholesterol (mmol/l)							
	Pre-CR (n=176)	1.1±0.3	1.2±0.4	1.1±0.3			
	Post-CR (n=187)	1.2±0.3	1.2±0.3	1.2±0.3	-0.1 (-0.2; -0.1)	-0.4 (-0.6; -0.2)	**<0.001**

	Descriptive statistics<br><br>Total (n=212)	Descriptive statistics<br><br>SIT LESS (n=108)	Descriptive statistics<br><br>Control (n=104)	Constrained mixed model<br><br>Time*group interaction (95% CI)	Constrained mixed model<br><br>Cohen's d (95% CI)	Constrained mixed model<br><br>p-value interaction
Triglycerides (mmol/l)						
Pre-CR (n=176)	1.4 [1.0 – 2.2]	1.4 [1.0 – 2.0]	1.5 [1.0 – 2.4]			
Post-CR (n=187)	1.4 [1.0 – 2.0]	1.4 [0.9 – 2.0]	1.5 [1.0 – 2.0]	0.0 (-0.2; 0.3)	0.0 (-0.2; 0.3)	0.83
Hemoglobin (mmol/l)						
Pre-CR (n=204)	8.6±1.0	8.5±1.0	8.6±1.0			
Post-CR (n=174)	8.7±0.8	8.7±0.7	8.6±0.9	0.1 (-0.1; 0.4)	0.1 (-0.1; 0.4)	0.26
Leucocytes (*10^9/l)						
Pre-CR (n=202)	8.6 [6.9 – 11.1]	8.6 [6.9 – 11.0]	8.5 [7.0 – 11.1]			
Post-CR (n=172)	6.8 [6.0 – 8.1]	6.6 [5.9 – 8.2]	7.0 [6.1 – 8.0]	-0.1 (-0.8; 0.6)	-0.0 (-0.3; 0.2)	0.73
Thrombocytes (*10^9/l)						
Pre-CR (n=202)	242±81	246±95	238±63			
Post-CR (n=172)	254±60	248±54	261±65	-16 (-35; 4)	-0.2 (-0.5; 0.0)	0.11

The control group is used as reference of the SIT LESS randomized controlled trial study outcomes. Descriptive statistics are depicted for the total group, as well as for the SIT LESS and control group separately. Descriptive statistics are presented as n (%) for categorical variables and as mean (± standard deviation) for normal distributed continuous data or median [interquartile range] for non-normal distributed continuous variables. Constrained linear mixed model outcomes are presented as unstandardized beta (95% Confidence interval (CI)) for the interaction group*time. CAD: coronary artery disease; HeartQoL: Heart quality of life. [a] Odds ratio with 95% CI.
