# Supplementary material for: A cardiac-rehab behaviour intervention to reduce sedentary time in coronary artery disease patients: the SIT LESS randomized controlled trial
Source: Int J Behav Nutr Phys Act. 2024 Aug 19;21:90. doi: 10.1186/s12966-024-01642-2 (PMC11331608; doi:10.1186/s12966-024-01642-2)
Supplement: Supplementary file 6 — Supplementary Figure 3: Constrained mixed model of the Timed up and go (TUG) (A) and Handgrip strength (HGS) (B). [file 12966_2024_1642_MOESM6_ESM.pdf]

A cardiac-rehab behaviour intervention to reduce sedentary time in coronary artery disease patients: The SIT LESS Randomized Controlled Trial

Sophie H. Kroesen, MSc[a]; Bram M.A. van Bakel, MD, PhD[a]; Marijn de Bruin, PhD[b]; Arzu Günal, MD[c]; Arko Scheepmaker, MD[c]; Wim R.M. Aengevaeren, MD, PhD[d]; Frank F. Willems, MD, PhD[d]; Roderick Wondergem, PhD[e,f,g]; Martijn F. Pisters, PhD[e,f,g]; Francisco B. Ortega, PhD[h,i,j]; Maria T.E. Hopman, MD, PhD[a]; Dick H.J. Thijssen, PhD[a,k]; Esmée A. Bakker, PhD[a,h,l]; Thijs M.H. Eijsvogels, PhD[a]

Affiliations:

[a] Radboud university medical center, Department of Medical BioSciences, Geert Grooteplein Zuid 10, 6525 GA, Nijmegen, The Netherlands.

[b] Radboud university medical center, Department of IQ healthcare, Geert Grooteplein Zuid 10, 6525 GA, Nijmegen, The Netherlands.

[c] Bernhoven hospital, Department of Cardiology, Nistelrodeseweg 10, 5406 PT, Uden, The Netherlands

[d] Rijnstate hospital, Department of Cardiology, Wagnerlaan 55, 6815 AD, Arnhem, The Netherlands

[e] Utrecht University, University Medical Centre Utrecht Brain Centre, Physical Therapy Science and Sport, Department of Rehabilitation, Universiteitsweg 100, 3584 CG, Utrecht, The Netherlands

[f] Fontys University of Applied Sciences, Department of Health Innovations and Technology, Research Group Empowering Healthy Behaviour, Rachelsmolen 1, 5612 MA, Eindhoven, The Netherlands

[g] Julius Health Care Centres, Centre for Physical Therapy Research and Innovation in Primary Care, Universiteitsweg 100, 3584 CG, Utrecht, the Netherlands

[h] University of Granada, Sport and Health University Research Institute (iMUDS), Department of Physical Education and Sports, Parque Tecnologico de la Salud, Av. del Conocimiento, s/n, 18007, Granada, Spain.

[i] CIBERobn Physiopathology of Obesity and Nutrition, Av. Monforte de Lemos, 3-5. Pabellón 11. Planta 0 28029, Madrid, Spain

[j] University of Jyväskylä, Faculty of Sport and Health Sciences, Keskussairaalantie 4, 40600, Jyväskylä, Finland

[k] Liverpool John Moores University, Research Institute for Sports and Exercise Sciences, Tom Reilly Building, Byrom Street, Liverpool, L3 3AF, United Kingdom

[l] Radboud university medical center, Department of Primary and Community Care, Geert Grooteplein Zuid 10, 6525 GA, Nijmegen, The Netherlands.

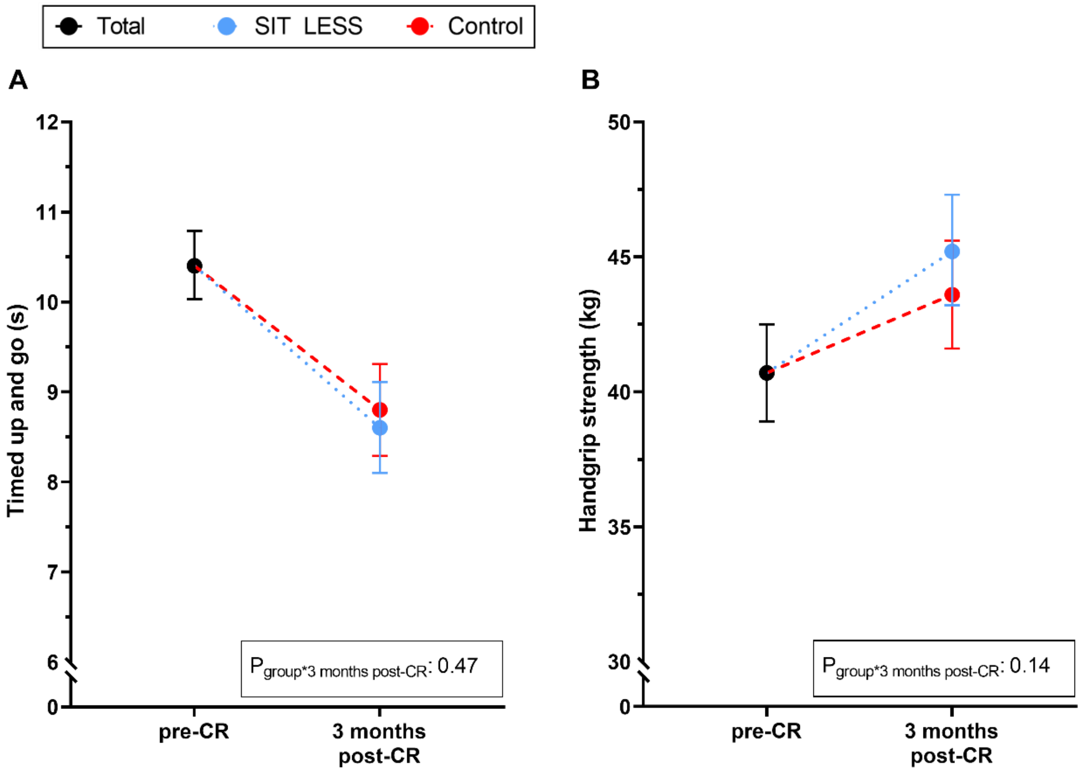

Supplementary Figure 3. Constrained mixed model of the Timed up and go (TUG) (A) and Handgrip strength (HGS) (B).

The total (black) group pre-cardiac rehabilitation (CR; TUG: SIT LESS: n=96; control: n=86. HGS: SIT LESS: n=73; control: n=76) and for the SIT LESS (blue) and control (red) 3 months post-CR (TUG: SIT LESS: n=92; control: n=92. HGS: SIT LESS: n=92; control: n=94) are depicted. Values are given as mean and 95% confidence interval.
