# Supplementary material for: A cardiac-rehab behaviour intervention to reduce sedentary time in coronary artery disease patients: the SIT LESS randomized controlled trial
Source: Int J Behav Nutr Phys Act. 2024 Aug 19;21:90. doi: 10.1186/s12966-024-01642-2 (PMC11331608; doi:10.1186/s12966-024-01642-2)
Supplement: Supplementary file 8 — Supplementary Document 1: TiDieR checklist. [file 12966_2024_1642_MOESM8_ESM.pdf]

TIDieR
Template for Intervention Description and Replication

The TIDieR (Template for Intervention Description and Replication) Checklist*:

Information to include when describing an intervention and the location of the information

**Item number**	**Item**	**Where located ****	
		Primary paper (page or append number)	Other † (details)
	**BRIEF NAME**		
**1.**	Provide the name or a phrase that describes the intervention.	P4	trial paper[1]<br>Short-term paper[2]
	**WHY**		
**2.**	Describe any rationale, theory, or goal of the elements essential to the intervention.	P5	trial paper[1]
	**WHAT**		
**3.**	Materials: Describe any physical or informational materials used in the intervention, including those provided to participants or used in intervention delivery or in training of intervention providers. Provide information on where the materials can be accessed (e.g. online appendix, URL).	P5	trial paper[1]
**4.**	Procedures: Describe each of the procedures, activities, and/or processes used in the intervention, including any enabling or support activities.	P5	trial paper[1]
	**WHO PROVIDED**		
**5.**	For each category of intervention provider (e.g. psychologist, nursing assistant), describe their expertise, background and any specific training given.		trial paper[1]<br>Short-term paper[2]

	**HOW**		
**6.**	Describe the modes of delivery (e.g. face-to-face or by some other mechanism, such as internet or telephone) of the intervention and whether it was provided individually or in a group.	P5	Short-term paper[2]
	**WHERE**		
**7.**	Describe the type(s) of location(s) where the intervention occurred, including any necessary infrastructure or relevant features.	P4	trial paper[1]
	**WHEN and HOW MUCH**		
**8.**	Describe the number of times the intervention was delivered and over what period of time including the number of sessions, their schedule, and their duration, intensity or dose.	P5	trial paper[1]<br>Short-term paper[2]
	**TAILORING**		
**9.**	If the intervention was planned to be personalised, titrated or adapted, then describe what, why, when, and how.	P5	trial paper[1]<br>Short-term paper[2]
	**MODIFICATIONS**		
**10.‡**	If the intervention was modified during the course of the study, describe the changes (what, why, when, and how).	NA	
	**HOW WELL**		
**11.**	Planned: If intervention adherence or fidelity was assessed, describe how and by whom, and if any strategies were used to maintain or improve fidelity, describe them.		Short-term paper[2]
**12.‡**	Actual: If intervention adherence or fidelity was assessed, describe the extent to which the intervention was delivered as planned.		Short-term paper[2]

** **Authors** - use N/A if an item is not applicable for the intervention being described. **Reviewers** – use '?' if information about the element is not reported/not sufficiently reported.

† If the information is not provided in the primary paper, give details of where this information is available. This may include locations such as a published protocol or other published papers (provide citation details) or a website (provide the URL).

‡ If completing the TIDieR checklist for a protocol, these items are not relevant to the protocol and cannot be described until the study is complete.

* We strongly recommend using this checklist in conjunction with the TIDieR guide (see *BMJ* 2014;348:g1687) which contains an explanation and elaboration for each item.

* The focus of TIDieR is on reporting details of the intervention elements (and where relevant, comparison elements) of a study. Other elements and methodological features of studies are covered by other reporting statements and checklists and have not been duplicated as part of the TIDieR checklist. When a **randomised trial** is being reported, the TIDieR checklist should be used in conjunction with the CONSORT statement (see www.consort-statement.org) as an extension of **Item 5 of the CONSORT 2010 Statement.** When a **clinical trial protocol** is being reported, the TIDieR checklist should be used in conjunction with the SPIRIT statement as an extension of **Item 11 of the SPIRIT 2013 Statement** (see www.spirit-statement.org). For alternate study designs, TIDieR can be used in conjunction with the appropriate checklist for that study design (see www.equator-network.org).

REFERENCES:

1. van Bakel BMA, Kroesen SH, Günal A, Scheepmaker A, Aengevaeren WRM, Willems FF, Wondergem R, Pisters MF, Dam J, Janssen AM, et al: **Sedentary Behaviour Intervention as a Personalised Secondary Prevention Strategy (SIT LESS) for patients with coronary artery disease participating in cardiac rehabilitation: rationale and design of the SIT LESS randomised clinical trial.** *BMJ Open Sport Exerc Med* 2022, **8:**e001364.

2. van Bakel BMA, Kroesen SH, Bakker EA, van Miltenburg RV, Günal A, Scheepmaker A, Aengevaeren WRM, Willems FF, Wondergem R, Pisters MF, et al: **Effectiveness of an intervention to reduce sedentary behaviour as a personalised secondary prevention strategy for patients with coronary artery disease: main outcomes of the SIT LESS randomised clinical trial.** *Int J Behav Nutr Phys Act* 2023, **20:**17.
