# Supplementary material for: A cardiac-rehab behaviour intervention to reduce sedentary time in coronary artery disease patients: the SIT LESS randomized controlled trial
Source: Int J Behav Nutr Phys Act. 2024 Aug 19;21:90. doi: 10.1186/s12966-024-01642-2 (PMC11331608; doi:10.1186/s12966-024-01642-2)
Supplement: Supplementary file 9 — Supplementary Document 2: Supplementary methods. [file 12966_2024_1642_MOESM9_ESM.pdf]

A cardiac-rehab behaviour intervention to reduce sedentary time in coronary artery disease patients: The SIT LESS Randomized Controlled Trial

Sophie H. Kroesen, MSc[a]; Bram M.A. van Bakel, MD, PhD[a]; Marijn de Bruin, PhD[b]; Arzu Günal, MD[c]; Arko Scheepmaker, MD[c]; Wim R.M. Aengevaeren, MD, PhD[d]; Frank F. Willems, MD, PhD[d]; Roderick Wondergem, PhD[e,f,g]; Martijn F. Pisters, PhD[e,f,g]; Francisco B. Ortega, PhD[h,i,j]; Maria T.E. Hopman, MD, PhD[a]; Dick H.J. Thijssen, PhD[a,k]; Esmée A. Bakker, PhD[a,h,l]; Thijs M.H. Eijsvogels, PhD[a]

Affiliations:

[a] Radboud university medical center, Department of Medical BioSciences, Geert Grooteplein Zuid 10, 6525 GA, Nijmegen, The Netherlands.

[b] Radboud university medical center, Department of IQ healthcare, Geert Grooteplein Zuid 10, 6525 GA, Nijmegen, The Netherlands.

[c] Bernhoven hospital, Department of Cardiology, Nistelrodeseweg 10, 5406 PT, Uden, The Netherlands

[d] Rijnstate hospital, Department of Cardiology, Wagnerlaan 55, 6815 AD, Arnhem, The Netherlands

[e] Utrecht University, University Medical Centre Utrecht Brain Centre, Physical Therapy Science and Sport, Department of Rehabilitation, Universiteitsweg 100, 3584 CG, Utrecht, The Netherlands

[f] Fontys University of Applied Sciences, Department of Health Innovations and Technology, Research Group Empowering Healthy Behaviour, Rachelsmolen 1, 5612 MA, Eindhoven, The Netherlands

[g] Julius Health Care Centres, Centre for Physical Therapy Research and Innovation in Primary Care, Universiteitsweg 100, 3584 CG, Utrecht, the Netherlands

[h] University of Granada, Sport and Health University Research Institute (iMUDS), Department of Physical Education and Sports, Parque Tecnologico de la Salud, Av. del Conocimiento, s/n, 18007, Granada, Spain.

[i] CIBERobn Physiopathology of Obesity and Nutrition, Av. Monforte de Lemos, 3-5. Pabellón 11. Planta 0 28029, Madrid, Spain

[j] University of Jyväskylä, Faculty of Sport and Health Sciences, Keskussairaalantie 4, 40600, Jyväskylä, Finland

[k] Liverpool John Moores University, Research Institute for Sports and Exercise Sciences, Tom Reilly Building, Byrom Street, Liverpool, L3 3AF, United Kingdom

[l] Radboud university medical center, Department of Primary and Community Care, Geert Grooteplein Zuid 10, 6525 GA, Nijmegen, The Netherlands.

SUPPLEMENTARY METHODS

Constrained linear mixed-model

Model

Primary and secondary outcome analysis were performed on an intention-to-treat basis using a constrained linear mixed-model analysis. A constrained (i.e. baseline adjusted) linear mixed-model can only be applied in randomized controlled trials as baseline imbalances are attributed to random chance and independent from the treatment[1]. In contrast to a linear mixed-model, a constrained linear mixed-model analysis assumes and fits a common baseline mean across randomized groups[2]. For a conventional linear mixed-model analysis, our model with two groups (1= control, 2= SIT LESS) and three time points (t=1, 2, 3) could be written as:

ST = intercept + group (=SIT LESS) + time2 + time 3 + group*time2 + group*time3 + (1 | patient)

Where the constrained-linear mixed model omits the effect as group to fit a common baseline mean. Therefore, the constrained-linear mixed model used in this study was written as:

ST = intercept + time2 + time 3 + group*time2 + group*time3 + (1 | patient)

Missing data

A constrained-linear mixed model is the optimal choice to assess intervention effects when data of either pre-CR or any of the post-CR measurements is missing[1, 2]. This model can handle missing data as both the pre-CR as the follow-up measurements are part of the outcome vector. Besides, the missing data should be related to observable characteristics (e.g. randomization group, time point of measuring) that are part of the model[2]. As the predictors of missing data are included in the mixed

model, the missing data can be considered as missing at random and the maximum likelihood estimation producer delivers unbiased estimates[3].

References

1. Liu GF, Lu K, Mogg R, Mallick M, Mehrotra DV: **Should baseline be a covariate or dependent variable in analyses of change from baseline in clinical trials?** *Stat Med* 2009, **28:**2509-2530.
2. Coffman CJ, Edelman D, Woolson RF: **To condition or not condition? Analysing 'change' in longitudinal randomised controlled trials.** *BMJ Open* 2016, **6:**e013096.
3. Olsen MK, Stechuchak KM, Edinger JD, Ulmer CS, Woolson RF: **Move over LOCF: Principled methods for handling missing data in sleep disorder trials.** *Sleep Med* 2012, **13:**123-132.
